# A Case Report of Accelerated Pulmonary Silicosis: Hazardous Exposure in Underrepresented Community Members

**DOI:** 10.7759/cureus.26587

**Published:** 2022-07-05

**Authors:** Sara Patino, Guillermo Izquierdo-Pretel

**Affiliations:** 1 Internal Medicine, Florida International University, Herbert Wertheim College of Medicine, Miami, USA

**Keywords:** immigrant health, eggshell calcification, pneumoconiosis, occupational exposure to silica, occupational lung disease, silicosis

## Abstract

A 39-year-old undocumented male immigrant with occupational exposure to quartz, without significant medical history, presented with worsening cough, dyspnea, and weight loss for the past six months, with onset of symptoms starting two years ago. The patient was exposed to silicon dioxide for the past 10 years due to his occupation. Chest x-ray at the time of diagnosis reflected multifocal heterogeneous opacities; CT of the chest demonstrated eggshell calcifications and conglomerate opacities particularly involving the bilateral upper lobes. Transbronchial biopsy reflected non-necrotizing granulomatous inflammation. In the context of silica dust exposure, these findings suggest the diagnosis of accelerated silicosis. This case report seeks to bring attention to the potential increased risk of occupational diseases in the community members who have high exposure to environmental hazards.

## Introduction

Silicosis refers to a spectrum of lung diseases caused by inhalation of free crystalline silica. Silicosis can be categorized as accelerated, acute, or chronic. Accelerated silicosis develops within five to 15 years of exposure. Cellular nodules composed of histiocytes, resembling granulomas, are a notable feature of accelerated silicosis due to heavy silica exposure [1].

Pulmonary silicosis is a preventable occupational hazard that can progress to respiratory failure and death. The empirical evidence available with regards to the relationship between illegal immigration status and occupational risk is narrow, partially due to data collection limitations. Although the overall mortality attributable to silicosis has lessened in the USA over the last 30 years due to improved workplace protections, this clinical case allows for the suggestion that there exists a gap in occupational protections for undocumented patients, leaving this population vulnerable to serious medical conditions. Occupational Safety and Health Administration (OSHA) does not mandate employers to provide protection to independent contractors from workplace hazards [2].

## Case presentation

A 39-year-old undocumented immigrant from Guatemala presented to the emergency department with complaints of progressive worsening cough, dyspnea, and weight loss over the last six months, with the onset of symptoms starting two years prior. Over time, his condition deteriorated, leading to constant fatigue and an unintentional 25-pound weight loss starting six months prior to his hospitalization. The patient continuously experienced cough with translucent white sputum production as well as dyspnea, sporadic fever, and night sweats. The patient was unaware of being in contact with tuberculosis-infected individuals and denied hemoptysis, rashes, or lymphadenopathy.

Past surgical history included left mandibular open fixation due to a fracture in 2012 and abdominal surgery due to stab wound in 2013. Family history was unremarkable. No medications or allergies were reported. The patient had a smoking history of 16 pack-years with smoking cessation occurring five years prior to his hospitalization. He reported minimal alcohol intake and denied illicit drug use. He had been working for the last 10 years in Miami, where there is high exposure to silicon oxide through his manual labor regarding stone cutting of quartz for fabrication of countertops. He reported that the respirator provided at this job allowed for dust particles to penetrate inside the mask. Proper mask fittings had never been performed. The employer had not offered the patient to complete medical examinations including chest x-rays or pulmonary function tests every three years despite the presence of respiratory symptoms or having the occupational need to wear a respirator for more than 30 days per year, as mandated by Occupational Safety and Health Administration (OSHA) standards [3]. The patient reported that a coworker employed at the same company for 18 years died three years ago with a similar clinical presentation.

**Figure 1:**
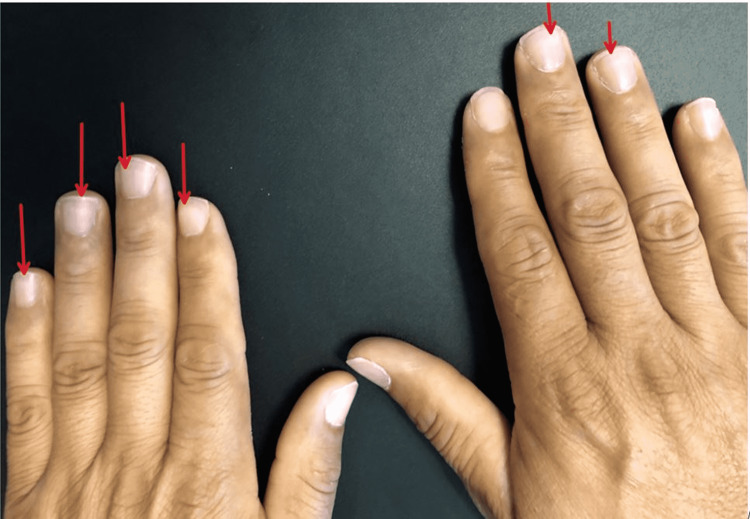
Mees' lines evidenced in physical examination associated with toxic exposure.

Physical examination findings included diffusely decreased breath sound bilaterally, with the right side being more affected compared to the left. Mild tracheal deviation to the right was present. No adventitious sounds were evident. Integumentary examination revealed evidence of Mees’ lines, which have been associated with occupational exposures (Figure 1).

Investigations

**Figure 2:**
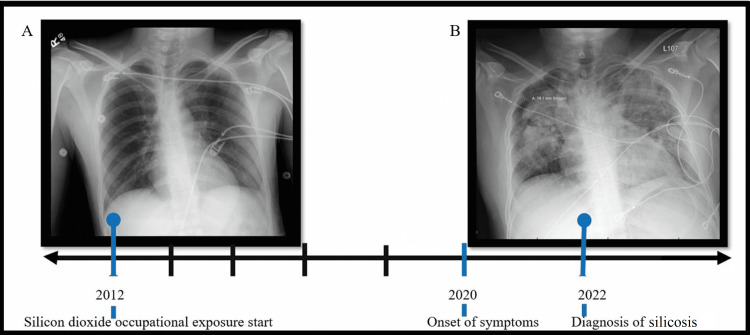
Chest x-ray transition regarding accelerated silicosis. The image shows (A) the chest x-ray 10 years prior and (B) the chest x-ray at the time of diagnosis.

A chest x-ray from 10 years ago was unremarkable. The chest x-ray gathered during the patient’s most recent admission demonstrated multifocal heterogeneous opacities of the bilateral lungs with a right apical pneumothorax. Figure 2 demonstrates the changes over time.

**Figure 3:**
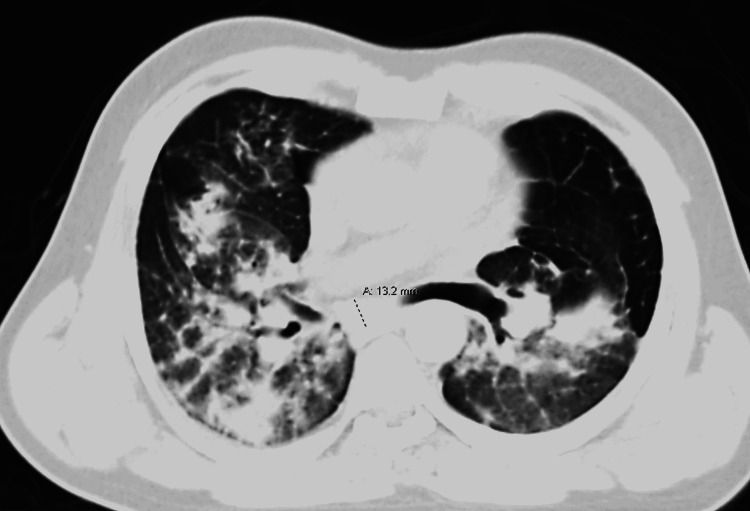
Chest CT without (w/o) contrast in the context of silicosis.

CT of the chest without contrast demonstrated mass-like conglomerate opacities in the upper lobes with associated radiating strands reflective of progressive massive fibrosis with superior hilar retraction (Figure 3). The CT imaging also portrayed extensive upper predominant bilateral pulmonary nodularity with peri-lymphatic distribution in the setting of calcified mediastinal and hilar lymphadenopathy. Right mild pneumothorax of approximately 30% was also visible.

Complete blood count (CBC) and comprehensive metabolic panel (CMP) were unremarkable; c-reactive protein was mildly elevated at 2 mg/dL. Workup for Aspergillus galactomannan antigen, Cryptococcus antigen, Histoplasma galactomannan antigen, blastomycosis, HIV, Legionella antigen, *Mycobacterium tuberculosis* (MTB) complex, *Pneumocystis carinii*, QuantiFERON-tuberculosis (TB) plus, Strep Pneumo antigen, and SARS-CoV-2 RNA reverse transcription-polymerase chain reaction (RT-PCR) were negative.

Immunological workup was unremarkable. Immunology workup included rheumatoid factor, antinuclear antibodies (ANA), antineutrophil cytoplasmic antibodies (ANCA), anti-dsDNA, anti-centromere antibody, anti-chromatic antibody, anti-Jo-1 antibody, anti-scleroderma 70 antibody, anti-ribosomal P antibody, anti-small ribonucleoprotein (SmRNP) antibody, anti-ribonucleoprotein (RNP), anti-Smith antibody, anti-Sjögren syndrome type B (SSB) antibody, and anti-Sjögren syndrome type A (SSA) antibody.

**Figure 4:**
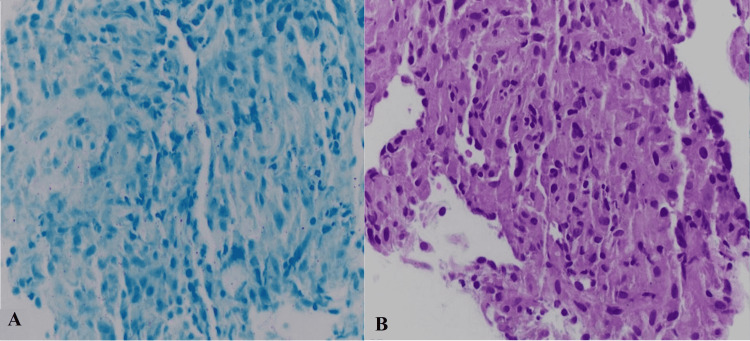
AFB stain, negative for acid-fast bacilli (A) and non-necrotizing granuloma 400x (B). AFB: acid-fast bacilli

Bronchoalveolar lavage was negative for malignancies and detected no evidence of viral cytopathic effect or fungal elements (Figure 4A). Figure 4B demonstrates the transbronchial biopsy of lung parenchyma with non-necrotizing granulomatous inflammation that, in the context of occupational exposure to quartz, confirms the diagnosis of silicosis.

Differential diagnosis

The patient’s presentation was concerning for lung malignancies, tuberculosis, fungal infections, autoimmune granulomatous diseases, and silicosis. Infectious diseases including fungal infections and TB were ruled out based on negative results for MTB PCR sputum, acid-fast bacilli (AFB) smear sputum, QuantiFERON-TB plus, and antigens for histoplasmosis, blastomycosis, and Cryptococcus. Transbronchial biopsy also did not find evidence of fungus or mycobacteria via implementation of special stains. Malignancies were ruled out based on the lack of evidence on CT of the chest and negative findings on the bronchoalveolar lavage. All markers for autoimmune diseases were negative in this patient. The following findings confirm the diagnosis of silicosis: (1) high exposure to silica dust for over 10 years with chest x-ray evidence of multifocal heterogeneous opacities, (2) CT of the chest demonstrating eggshell calcifications and conglomerate opacities particularly involving the bilateral upper lobes, and (3) transbronchial biopsy reflecting non-necrotizing granulomatous inflammation.

Patient's perspective

The patient understands that as an undocumented immigrant, the legal action or compensation that he can pursue due to occupational hazards is limited. His wish is to continue working in another field despite his current vulnerable condition in order to provide for his wife and two young children.

## Discussion

The clinical presentation of silicosis can vary. Silicosis may present as an acute, chronic, or accelerated form. This patient with a 10-year exposure to silicon dioxide via Quartz manipulation presented with the accelerated form of silicosis. Limited case report publications exist representing a possible increased link between undocumented immigrants and occupational diseases. The case report therefore presents an accelerated case of silicosis in an undocumented patient in the United States.

Silica in the work environment can be found in occupations including mining fracturing, sandblasting, foundry work, masonry, and stone cutting. The inhalation of the silica dust deposits in the airways, effectuating oxygen radicals with the crystalline silica combines with the aqueous media of the lungs. The subsequent inflammatory reaction that occurs leads to pulmonary scarring and fibrosis [4]. The accelerated version of silicosis overlaps with features from acute and chronic diseases. Dyspnea on exertion and chronic cough are the most common symptoms, which were primary symptoms present in the patient. Although the physical examination may reflect adventitious sounds, usually chest examination is unremarkable. Diagnostic investigation can comprise pulmonary function tests typically illustrating decreased lung volumes and diffusion capacity. Other diagnostic investigations can be with imaging such as chest x-rays revealing ground glass opacities or high-resolution CT demonstrating bilateral centrilobular nodular opacities and patchy areas of consolidation. The diagnosis of silicosis can be made without lung biopsy [5]. Nevertheless, it can help confirm a case of silicosis, as was done in the case of the patient discussed.

Treatment and prognosis

No specific therapy has been identified for silicosis. The treatment for this condition revolves around avoidance of further silicon dioxide exposure - either by change of occupation or by optimization of protective equipment with the minimum requirements as established by OSHA - and supportive care. OSHA describes the permissible exposure limit (PEL) for respirable silica to be 50 μg/m^3^ over an eight-hour shift. National Institute for Occupational Safety and Health (NIOSH) recommends the use of N95 masks for those cases below 50 μg/m^3^. Otherwise, powered respirators are recommended. Supportive therapy includes supplemental oxygenation if indicated, treatment of airflow limitations with bronchodilators, and vaccination against pneumococcus and influenza [5].

The poor prognosis, with survival of less than four years after onset of symptoms, has prompted experimental therapy with glucocorticoids and whole lung lavage. However, formal evaluation of these experiments has not been done. Moreover, glucocorticoid therapy has effectuated the development of progressive massive fibrosis. Lung transplantation is an option for patients with respiratory failure [5]. However, the latter option is limited for undocumented immigrants.

## Conclusions

High exposure to respirable silica poses a risk for development of silicosis, presenting with constitutional and respiratory symptoms usually after 10 years of exposure. Accelerated silicosis develops in less time with presence of non-necrotizing granulomas. The diagnosis for silicosis is made based on occupational history, clinical presentation, and diagnostic investigations such as chest x-ray findings of eggshell calcifications and ground glass opacities especially affecting the upper lobes of the lungs. A lung biopsy can be done only to rule out other associated pathology. Protective measures including N95 masks, respirators, or medical surveillance are to be taken to prevent the development of silicosis when occupational exposure to silica dust exists. Undocumented immigrants may be at increased risk for developing occupational diseases, partially due to the lack of requirements for employers to abide by OSHA standards when dealing with independent contractors, including undocumented immigrants, as well as need for undocumented immigrants to pursue less desired occupations.
